# SARS-CoV-2 vaccines are not associated with hypercoagulability in apparently healthy people

**DOI:** 10.1016/j.rpth.2022.100002

**Published:** 2022-11-25

**Authors:** Lamya Garabet, Anna Eriksson, Eirik Tjønnfjord, Xue-Yan Cui, Magnus Kringstad Olsen, Hege Karine Jacobsen, Camilla Tøvik Jørgensen, Åse-Berit Mathisen, Marie-Christine Mowinckel, Maria Therese Ahlen, Ingvild Hausberg Sørvoll, Kjersti Daae Horvei, Siw Leiknes Ernstsen, Ingvild Jenssen Lægreid, Benedicte Stavik, René Holst, Per Morten Sandset, Waleed Ghanima

**Affiliations:** 1 Center for Laboratory Medicine, Østfold Hospital, Grålum, Norway; 2 Multidisciplinary Laboratory Medicine and Medical Biochemistry, Akershus University Hospital, Nordbyhagen, Norway; 3 Department of Research, Østfold Hospital, Grålum, Norway; 4 Department of Medicine, Østfold Hospital, Grålum, Norway; 5 Department of Haematology, Oslo University Hospital, Oslo, Norway; 6 Research Institute of Internal Medicine, Oslo University Hospital, Oslo, Norway; 7 Department of Emergency Medicine, Østfold Hospital, Grålum, Norway; 8 Norwegian National Unit for Platelet Immunology, Department of Laboratory Medicine, University Hospital of North Norway, Tromsø, Norway; 9 Department of Medical Biology, University of Tromsø, Tromsø, Norway; 10 Institute of Clinical Medicine, University of Oslo, Oslo, Norway

**Keywords:** coagulation activation, D-dimer, hypercoagulable state, SARS-CoV-2 vaccines, thrombin generation, thrombocytopenia

## Abstract

**Background:**

SARS-CoV-2 adenoviral vector DNA vaccines have been linked to the rare but serious thrombotic postvaccine complication vaccine-induced immune thrombotic thrombocytopenia. This has raised concerns regarding the possibility of increased thrombotic risk after any SARS-CoV-2 vaccines.

**Objectives:**

To investigate whether SARS-CoV-2 vaccines cause coagulation activation leading to a hypercoagulable state.

**Methods:**

This observational study included 567 health care personnel; 521 were recruited after the first dose of adenoviral vector ChAdOx1-S (Vaxzevria, AstraZeneca) vaccine and 46 were recruited prospectively before vaccination with a messenger RNA (mRNA) vaccine, either Spikevax (Moderna, n = 38) or Comirnaty (Pfizer-BioNTech, n = 8). In the mRNA group, samples were acquired before and 1 to 2 weeks after vaccination. In addition to the prevaccination samples, 56 unvaccinated blood donors were recruited as controls (total n = 102). Thrombin generation, D-dimer levels, and free tissue factor pathway inhibitor (TFPI) levels were analyzed.

**Results:**

No participant experienced thrombosis, vaccine-induced immune thrombotic thrombocytopenia, or thrombocytopenia (platelet count <100 × 10^9^/L) 1 week to 1 month postvaccination. There was no increase in thrombin generation, D-dimer level, or TFPI level in the ChAdOx1-S vaccine group compared with controls or after the mRNA vaccines compared with baseline values. Eleven of 513 (2.1%) participants vaccinated with ChAdOx1-S had anti-PF4/polyanion antibodies without a concomitant increase in thrombin generation.

**Conclusion:**

In this study, SARS-CoV-2 vaccines were not associated with thrombosis, thrombocytopenia, increased thrombin generation, D-dimer levels, or TFPI levels compared with baseline or unvaccinated controls. These findings argue against the subclinical activation of coagulation post-COVID-19 vaccination.

## Essentials

• Concerns have been raised regarding the risk of developing blood clots after COVID-19 vaccines.
• Thrombin generation was measured in 521 participants vaccinated with adenoviral vector and 46 participants vaccinated with a messenger RNA vaccine.
• Thrombin generation, which reflects the clotting tendency, did not increase after vaccination.
• COVID-19 vaccines do not seem to increase the risk of blood clots in healthy individuals.

## 1 Introduction

Vaccination against SARS-CoV-2 is still the most important measure to combat COVID-19. However, the anti–SARS-CoV-2 adenovirus-vectored vaccines, initially the ChAdOx1-S vaccine (Vaxzevria, AstraZeneca) and later the Janssen Ad26.COV2.S vaccine (Johnson & Johnson) have been linked to the development of a severe, life-threatening thromboembolic complication with thrombocytopenia—a condition termed vaccine-induced immune thrombotic thrombocytopenia (VITT) because of the formation of antibodies against platelet factor 4 (PF4) that activates platelets via the Fcγ receptors [[1], [2], [3]]. Early case reports on VITT came from Germany/Austria (11 cases) [1], Norway (5 cases) [2], and the United Kingdom (23 cases) [4]. Later, vaccine safety reports from the United Kingdom revealed >260 VITT cases between December 2020 and May 2021 following 30.8 million doses of the ChAdOx1-S vaccine for a risk rate of approximately 1 per 100,000 in those aged >50 years and 1 per 50,000 for those aged between 18 and 49 years. In Norway, the incidence was higher and was estimated to be 1 per 22,000, and for Norway and Denmark together, the incidence was 1 per 40,000 [5]. VITT occurred in apparently healthy individuals with no known thrombotic risk factors. VITT has been characterized by venous thrombosis at atypical sites, such as cerebral venous sinus thrombosis and splanchnic vein thrombosis, along with thrombocytopenia [1,2,4], clinically mimicking heparin-induced thrombocytopenia [1]. In addition to thrombocytopenia, these patients had elevated D-dimer, often decreased fibrinogen, and high levels of anti-PF4 antibodies that activated platelets independent of heparin [6]. It has been suggested that vaccine components form complexes with PF4, which in combination with vaccine-induced inflammation, trigger the formation of anti-PF4 antibodies. Those antibodies activate platelets and stimulate neutrophils to release neutrophil extracellular taps leading to coagulation activation and thrombosis [7,8]. In a large population-based cohort study, Whiteley et al. [9] found higher rates of intracranial venous thrombosis in ChAdOx1-S–vaccinated adults aged <70 years than in the unvaccinated adults, whereas the rate of major arterial and venous thrombosis was not increased in adults aged ≥70 years after both ChAdOx1-S and Pfizer-BioNTech vaccines.

These serious and potentially fatal complications after vaccination with the SARS-CoV-2 adenovirus-vectored vaccines have raised considerable concerns regarding whether the SARS-CoV-2 vaccines are generally associated with the development of autoantibodies leading to platelet activation, thrombocytopenia, and coagulation activation. The prevalence of anti-PF4/polyanion antibodies in 492 Norwegian health care personnel (also included in the current study) after receipt of the first dose of ChAdOx1-S vaccine has been previously published [10]. Anti-PF4/polyanion antibodies were detected by immunoassay only in 6 of 492 (1.2%) individuals (cutoff optical density, ≥0.400). None of the samples with anti-PF4/polyanion antibodies activated platelets, which is typical for VITT, and all had normal platelet counts.

In this study, we aimed to explore whether SARS-CoV-2 vaccines are associated with subclinical coagulation activation leading to a hypercoagulable state in apparently healthy vaccinated subjects.

## 2 Materials and methods

### 2.1 Study subjects

Health care personnel were recruited from 2 hospitals in Norway—the University Hospital of North Norway (UNN), Tromsø, and the Østfold Hospital, Kalnes—during March and April 2021. We included health care personnel who had received their first dose of ChAdOx1-S vaccine within 35 days and those who had persistent symptoms possibly related to the vaccine (mainly bleeding tendency and/or fatigue) beyond 35 days. This was to include those in whom the platelets and/or the coagulation system could have been affected, making a total of 521 participants. Therefore, the time interval following the vaccine varied from 11 to 57 days. The study was initiated after the occurrence of VITT in 5 health care personnel in Norway short time after receiving their first dose of ChAdOx1-S and the vaccine was put on hold; therefore, no baseline samples were available for these individuals. Health care personnel who were scheduled to receive a messenger RNA (mRNA) vaccine (n = 46), either elasomeran (Spikevax, Moderna, n = 38) or tozinameran (Comirnaty, Pfizer-BioNTech, n = 8), were recruited prospectively. In the prospective group, blood samples were acquired before and 1 to 2 weeks after vaccination. All participants signed a written informed consent. Fifty-six unvaccinated healthy blood donors from the blood donation service at UNN, with no history of COVID-19, were recruited as controls. In addition, the 46 health care personnel in the prospective group who donated blood before receiving mRNA vaccines also acted as controls making a total of 102 controls. The study was approved by the Norwegian research ethics committees (REK 257384 and REK 255184).

### 2.2 Clinical data and blood sampling

All participants completed a questionnaire to describe any side effect after vaccination such as fever, headache, bleeding tendency, cutaneous bleeding, fatigue, and muscle and joint pain. Blood samples were collected in EDTA tubes (Greiner Bio-One) for the analysis of platelet counts, which was performed on a Sysmex hematology analyzer (Sysmex) at UNN and on an ADVIA hematology analyzer (Siemens Healthineers) at Østfold Hospital. Blood samples were also collected in tubes containing 3.2% sodium citrate (Greiner Bio-One) that were centrifuged for 20 minutes at 2000 g at room temperature within 1 hour to prepare platelet poor plasma, and aliquots were stored at −80°C until analyzed.

### 2.3 Laboratory analysis

#### 2.3.1 Thrombin generation

Thrombin generation was assayed using the Calibrated Automated Thrombogram assay (Diagnostica Stago). In short, coagulation was triggered by recalcification of citrated plasma in the presence of an intermediate number of phospholipids and 5 pM tissue factor using PPP-Reagent and fluorogenic substrate, obtained from Thrombinoscope BV, as described by the manufacturer. We evaluated the following parameters of the thrombogram: lag time (time in minutes that follows the addition of the trigger until the initiation of thrombin generation); peak (represents the highest thrombin concentration [nmol] that has been generated); time to peak (ttPeak) (time it takes to reach the peak in minutes) and represents the velocity of thrombin generation); velocity index (VI) (calculated as peak height/[ttPeak − lag time]); and endogenous thrombin potential (ETP), which is calculated from the area under the curve (nmol thrombin × minutes) and represents the total amount of thrombin generated in the sample. The results of these parameters were normalized against pooled normal plasma measured in the same run and given as a ratio in percentage. A short lag time, high peak, short ttPeak, high VI, and high ETP reflect hypercoagulability, whereas a long lag time, low peak, long ttPeak, low VI, and low ETP reflect hypocoagulability [11].

#### 2.3.2 D-dimer

D-dimer was assayed using Asserachrom D-Dimer (Diagnostica Stago). D-dimer is generated when cross-linked fibrin polymers are degraded by plasmin; thus, elevated levels indicate both coagulation activation and fibrinolysis. The cutoff was <500 ng/mL fibrinogen equivalent units.

#### 2.3.3 Free tissue factor pathway inhibitor antigen

Free tissue factor pathway inhibitor antigen (TFPI) was assayed with Asserachrom Free TFPI (Diagnostica Stago). Most of the TFPI is bound to endothelial cells, but it is also found in platelets and is released on activation. TFPI in plasma is found in the following 2 forms: lipoprotein-associated (approximately 80%) and free TFPI. Only the free form of TFPI is associated with the anticoagulant activity. The normal level of TFPI is 10 ng/mL.

#### 2.3.4 Anti-PF4/polyanion antibodies

Screening for anti-PF4/polyanion antibodies was performed in 513 ChAdOx1-S–vaccinated health care personnel (8 were missing) using LIFECODES PF4 immunoglobulin G enzyme-linked immunosorbent assay (Immucor), with a cutoff value of ≥0.400 optical density. Screening for anti-PF4/polyanion antibodies was not performed in those vaccinated with mRNA vaccine. Platelet function test, heparin-induced multiple electrode aggregometry, was performed, and the results have been previously published [10]. Thus, these results would not be reported here.

### 2.4 Statistics

Continuous variables are presented as means, and the binary variable “sex” is presented as a proportion. The analyses included 3 comparisons: a comparison between the ChAdOx1-S–vaccinated individuals and unvaccinated controls, an intraindividual comparison of the pre- and postvaccination values of the mRNA-vaccinated individuals, and a comparison between those with anti-PF4/polyanion antibodies and those without anti-PF4/polyanion antibodies within the ChAdOx1-S–vaccinated individuals. These were all achieved by age- and sex-adjusted regressions, except for the anti-PF4/polyanion antibodies analysis, which only allowed for age adjustment. The analyses revealed varying dependencies of these variables. The pre- and postcomparisons used the within-individual changes as response variables, and the assumption of normality was found justified. The 2 other sets of regressions were conducted on transformed scales following the Box-Cox transformations to achieve normality. The results are presented on the original scale after back transformations and predicted for mean ages and with equal gender effects. The analyses were performed using R, version 4.2.0, and Stata version 17.

## 3 Results

In this study, we included 521 health care personnel vaccinated with ChAdOx1-S; 46 vaccinated with mRNA vaccines, either with Spikevax (Moderna, n = 38) or Comirnaty (Pfizer-BioNTech, n = 8); and 102 unvaccinated controls. None of the participants developed thrombosis, thrombocytopenia, or VITT after vaccination. The characteristics of the study population are presented in Table 1.

**Table 1:** Characteristics of the study populations.

	ChAdOx1-S	mRNA	Controls
No. of participants	521	46	102
Age, y	44 (21-69)	48 (25-66)	46 (22-66)
Female, %	76	67	50
Time since vaccination, d	20 (11-57)	11 (7-18)	—
Platelet count, 10^9^/L (baseline)	270 (101-490)	243 (133-365)	252 (135-549)

Data are presented as means (minimum-maximum).

### 3.1 Comparisons between the ChAdOx1-S vaccine group and the controls

The ETP and peak were lower and the lag time was longer in the ChAdOx1-S–vaccinated individuals, reflecting lower thrombin generation than in the controls. However, the ttPeak was shorter and VI was higher in the ChAdOx1-S–vaccinated individuals than in the controls. No difference in the levels of D-dimer or TFPI between the ChAdOx1-S–vaccinated individuals and the controls (Table 2) was observed. The differences in the means (95% CIs) are shown in the Figure.

**Table 2:** Mean values (95% CIs) for the different parameters after ChAdOx1-S vaccination and in controls, and difference in the means (95% CIs).

Studied parameters	ChAdOx1-S (n = 521)	Controls (n = 102)	Difference in means
Lag time, %	109 (107; 111)	103 (100; 107)	5.2 (1.1; 9.4)
Peak, %	82.4 (80.1; 84.8)	90.3 (85.2; 95.3)	−7.8 (−13.4; −2.2)
ttPeak, %	71.2 (67.9; 74.4)	81.7 (74.2; 89.3)	−10.5 (−18.7; −2.3)
ETP, %	91.8 (90; 93.5)	98.5 (94.8; 102)	−6.7 (−10.8; −2.6)
VI, %	113 (111; 115)	108 (104; 111)	5.4 (1.3; 9.6)
D-dimer, ng/mL	214 (205; 224)	227 (206; 248)	−13 (−36; 11)
TFPI, ng/mL	12.9 (12.6; 13.3)	12.6 (11.9; 13.3)	0.3 (−0.4; 1.1)

ETP, endogenous thrombin potential; TFPI, tissue factor pathway inhibitor; ttPeak, time to peak; VI, velocity index.

**Figure:**
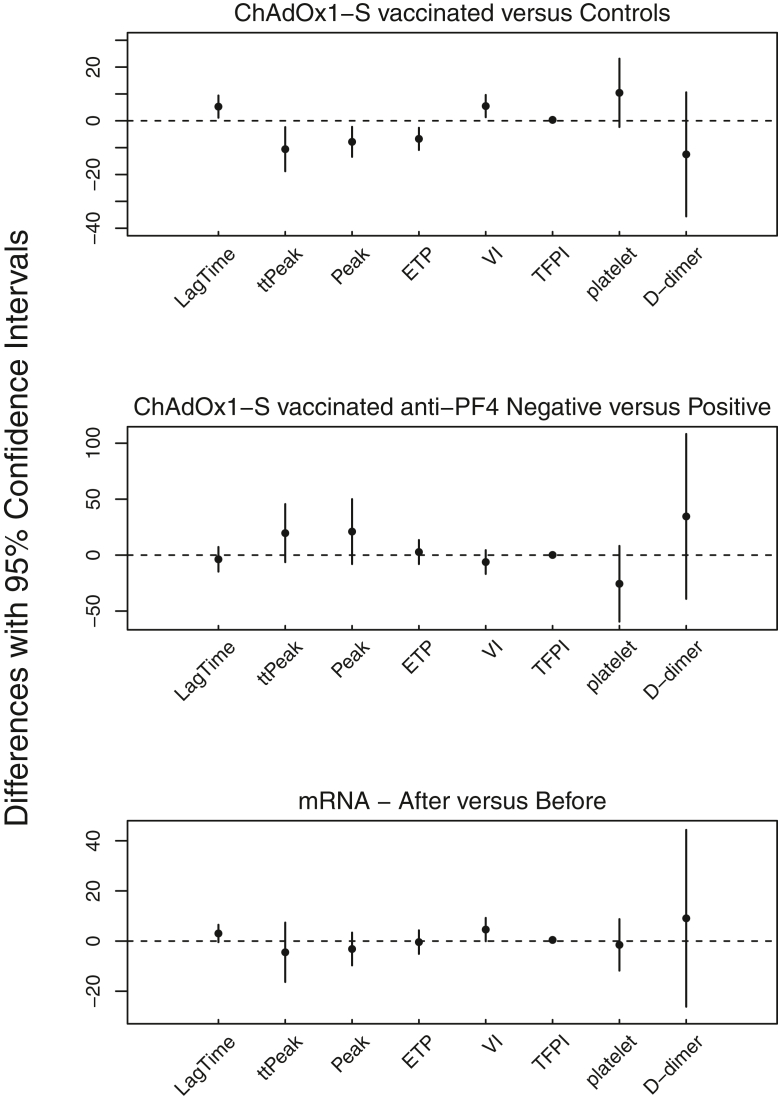
The mean difference with 95% CI of the difference for the 3 different comparisons PF4, platelet factor 4

### 3.2 Comparison between ChAdOx1-S–vaccinated individuals with and without anti-PF4/polyanion antibodies

Eleven of 513 (2.1%) individuals vaccinated with the ChAdOx1-S vaccine had anti-PF4/polyanion antibodies (optical density ≥0.400). None of the controls had anti-PF4/polyanion antibodies (all had optical density <0.200). No differences in the parameters of thrombin generation, D-dimer levels, or TFPI levels were found between the ChAdOx1-S–vaccinated individuals with anti-PF4/polyanion antibodies and those without anti-PF4/polyanion antibodies (Table 3). The differences in the means (95% CIs) are shown in the Figure.

**Table 3:** Mean values (95% CIs) of the different parameters for the ChAdOx1-S–vaccinated individuals with and without anti-PF4/polyanion antibodies, and the difference in the means (95% CIs).

Studied parameters	Anti-PF4 antibodies negative (n = 502)	Anti-PF4 antibodies positive (n = 11)	Difference in means
Lag time, %	106 (104; 108)	102 (91.9; 113)	−3.7 (−14.7; 7.2)
Peak, %	139 (135; 143)	160 (131; 189)	21.0 (−7.9; 50)
ttPeak, %	75.0 (71.9; 78.1)	94.7^a^ (68.9; 120)	19.6 (−6.2; 45.5)
ETP, %	92.9 (91.4; 94.4)	95.6 (85; 106)	2.7 (−7.9; 13.4)
VI, %	110 (109; 112)	104 (94.1; 115)	−6.1 (−16.7; 4.4)
D-dimer, ng/mL	226 (217; 235)	260 (187; 333)	35 (−39; 108)
TFPI, ng/mL	12.2 (11.9; 12.5)	12.3 (10.3; 14.4)	0.1 (−1.9; 2.2)

ETP, endogenous thrombin potential; TFPI, tissue factor pathway inhibitor; ttPeak, time to peak; VI, velocity index.

a n = 10; one outlier was removed.

### 3.3 Comparisons between before and after mRNA vaccines

There were no changes in the parameters of thrombin generation or in the D-dimer or TFPI levels after vaccination with mRNA vaccines compared with levels before vaccination (Table 4 and Figure).

**Table 4:** Mean values (95% CIs) for the different parameters before and after mRNA vaccination, and the difference in the means (95% CIs).

Studied parameters	Before mRNA (n = 46)	After mRNA (n = 46)	Difference in means (95% CI)
Lag time, %	111 (105; 116)	115 (109; 121)	3.0 (−0.4; 6.4)
Peak, %	82.3 (72.8; 91.9)	80.0 (70.4; 89.5)	−3.1 (−9.6; 3.3)
ttPeak, %	69.2 (55.2; 83.4)	65.7 (51.9; 79.4)	−4.4 (−16.3; 7.3)
ETP, %	96.4 (89.0; 103)	95.9 (88.4; 103)	−0.4 (−5.1; 4.2)
VI, %	115 (108; 123)	119 (111; 128)	4.5 (−0.05; 9.2)
D-dimer, ng/mL	242^a^ (207; 276)	249 (213; 284)	9 (−26; 44)
TFPI, ng/mL	12.5 (11.5; 13.5)	13.0 (12.0; 14.1)	0.4 (−0.2; 1.1)

ETP, endogenous thrombin potential; TFPI, tissue factor pathway inhibitor; ttPeak, time to peak; VI, velocity index.

a n = 45; one outlier was removed.

## 4 Discussion

In this observational study, there were no cases of thrombosis, thrombocytopenia, VITT, or *in vitro* evidence of coagulation activation after the SARS-CoV-2 vaccination in an apparently healthy population. There was no increase in thrombin generation, D-dimer levels, or TFPI levels in SARS-CoV-2–vaccinated individuals compared with unvaccinated controls. In contrast to patients with VITT, who typically have extremely high levels of D-dimer, the D-dimer levels in this apparently healthy population after ChAdOx1-S or mRNA vaccination were within the normal range and comparable with the levels in the unvaccinated controls. Eleven of 513 (2.1%) health care personnel vaccinated with ChAdOx1-S, but none of the controls, had anti-PF4/polyanion antibodies; however, the values were much lower than those seen in patients with VITT [10]. Importantly, these anti-PF4/polyanion antibodies found in vaccinated individuals were not associated with higher thrombin generation than those without anti-PF4/polyanion antibodies. However, the number of vaccinated individuals with anti-PF4/polyanion antibodies was small; therefore, the results should be interpreted with caution.

In line with these findings, 2 other studies that investigated the effect of SARS-CoV-2 vaccines on coagulation activation and thrombin generation found no significant increase. The first study evaluated 30 subjects before and after they received the Pfizer-BioNTech vaccine (7 and 21 days after the first dose and 14 days after the second dose) and found neither evidence of coagulation activation nor an increase in thrombin generation after vaccination [12]. The second study included 101 subjects who received the ChAdOx1-S vaccine and 89 who received the Pfizer-BioNTech vaccine without evidence of coagulation activation [13]. Conversely, a newly published Danish study that included 80 vaccinated individuals (55 ChAdOx1-S and 25 mRNA) found higher thrombin generation after ChAdOx1-S vaccine than after mRNA vaccines [14]. However, our study included more individuals vaccinated with the ChAdOx1-S vaccine (521 vs 55) and also included individuals with persistent possible vaccine-related symptoms up to 57 days after vaccination and, therefore, a wider range of time following the vaccine (11-57 days vs 8-16 days) without a significant increase in thrombin generation.

In addition to thrombin generation, we analyzed the levels of D-dimer, which reflects *in vivo* coagulation activation and ongoing fibrinolysis, and of TFPI, an inhibitor of the extrinsic coagulation pathway reflecting endothelial (and platelet) activation. These 2 parameters have been shown to be increased in critically ill patients with COVID-19 [15]; however, in line with thrombin generation, no differences were found between the vaccinated individuals and unvaccinated controls. A study from Thailand also showed no difference in D-dimer levels between ChAdOx1-S–vaccinated individuals and unvaccinated controls; however, in contrast to our study, the prevalence of anti-PF4 antibodies was comparable between the vaccinated individuals and unvaccinated controls [16].

A limitation of this study is that the size of the control group was smaller than that of the ChAdOx1-S vaccine group and was equally represented by both sexes, whereas there were more females in the vaccinated group; nevertheless, we adjusted for sex in the comparisons. Although females may have higher thrombin generation because of the use of oral contraceptives or hormone substitution, thrombin generation was not higher in the vaccine groups than in the controls. In addition, 55% of the controls in our study were unvaccinated healthy blood donors. We cannot exclude that these were generally healthier population than the vaccinated health care personnel. However, we have also included unvaccinated health care personnel at baseline as controls (45% of the controls), which would minimize the difference in comorbidity between the vaccinated individuals and controls. There was a difference in observation time interval following vaccination between the ChAdOx1-S vaccine group and the mRNA vaccine group, with a longer postvaccination observation window for the ChAdOx1-S vaccine group. We acknowledge that it would have been preferable to evaluate participants for an extended period after exposure to both vaccines to allow for an opportunity to observe for delayed postvaccine complications. Another limitation of this study is that the number of vaccinated individuals with anti-PF4/polyanion antibodies was very small compared with those without antibodies, limiting the robustness of these findings. Furthermore, other potential biomarkers of thrombotic risk, including prothrombin fragment 1+2, protein C resistance, or factor VIII, were not analyzed. We chose to perform the thrombin generation assay, which is a well-established global assay that provides a comprehensive picture on plasma coagulability and integrates all pro- and anticoagulant reactions that regulate the formation and inhibition of thrombin. Finally, the lack of information regarding sociocultural status, including race/ethnicity, could have affected the results; however, we anticipate that the participants were predominantly Caucasians.

In conclusion, our results are reassuring because they suggest that SARS-CoV-2 vaccines are neither generally associated with thrombotic events or thrombocytopenia nor with coagulation activation in apparently healthy individuals. Furthermore, the lack of association between anti-PF4/polyanion antibodies and coagulation activation is particularly reassuring and supports the need for a multihit inflammatory model in VITT.
